# Genetic evidence supports primary biliary cholangitis as a risk factor for lacunar stroke

**DOI:** 10.1016/j.isci.2025.114240

**Published:** 2025-11-27

**Authors:** Mengmeng Wang, Nan Zhang, Ling Zhang, Ning Zhang, Haichu Yu

**Affiliations:** 1 Department of Cardiology, The Affiliated Hospital of Qingdao University, Qing Dao, Shandong, China; 2 Department of Infectious Diseases, The Third Affiliated Hospital of Sun Yat-sen University, Guangzhou, Guangdong, China; 3 General Medicine Department, Heze Municipal Hospital, Heze, Shandong, China; 4 Institute of Cardiovascular Disease, Qingdao University, Qingdao 266001, Shandong, China

**Keywords:** Health sciences

## Abstract

Primary biliary cholangitis (PBC) is a chronic autoimmune liver disease, while lacunar stroke is a common cerebrovascular subtype. Understanding potential systemic vascular consequences of PBC is crucial for patient management. Using a two-sample Mendelian randomization (MR) approach, we investigated the causal effect of PBC on lacunar stroke risk. We utilized genetic instruments for PBC from large-scale genome-wide association studies and tested their association with lacunar stroke in independent datasets. In the discovery analysis, genetically predicted PBC was associated with an increased risk of lacunar stroke (odds ratio [OR] = 1.0635, 95% confidence interval [CI]: 1.0224–1.1063; *p* = 0.0022). This finding was consistently replicated using two updated PBC datasets (OR = 1.0671, *p* = 0.0014; OR = 1.0548, *p* = 0.0026). Sensitivity analyses indicated no significant pleiotropy and confirmed result robustness. This genetic evidence suggests PBC is a causal risk factor for lacunar stroke, warranting further investigation and targeted prevention.

## Highlights

• PBC causally increases risk of lacunar stroke
• MR evidence is consistent across multiple GWAS
• No horizontal pleiotropy detected in analyses
• Supports early stroke prevention in PBC patients

## Graphical abstract

**Figure:**
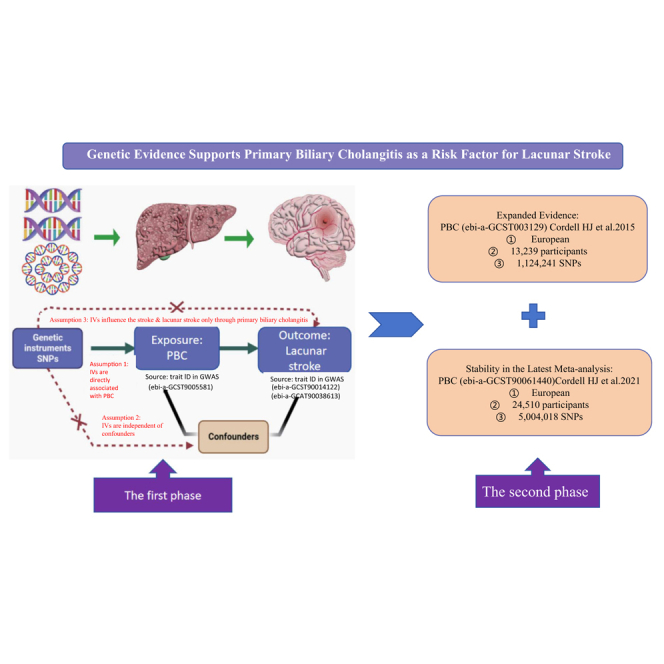

## Introduction

Primary biliary cholangitis (PBC) is a chronic disease characterized by the deterioration of small bile ducts within the liver, leading to inflammation around the portal areas, loss of bile ducts, impaired bile flow, and the development of biliary cirrhosis over time.^1^ PBC is considered a model autoimmune illness due to the serological findings, evidence of antimitochondrial antibody (AMA), and the unique bile duct pathological alterations observed in the disease.^2^ PBC is most commonly diagnosed when regular laboratory tests show an increase in alkaline phosphatase. AMA, the serological marker of PBC, is found in approximately 95% of people with the disease yet occurs in less than 1% of healthy adults. High alkaline phosphatase levels along with the presence of AMA are sufficient to identify the condition.^3^ PBC is more commonly diagnosed in women, with a female to male ratio of roughly 10 to 1.^4^ The prevalence of PBC ranges from 1.91 to 40.20 per 100,000 people and has grown over time.^5^ The clinical manifestations of PBC include pruritus, fatigue, and, less frequently, complications of jaundice or cirrhosis. The clinical manifestations and complications of PBC seriously affect the quality of life of patients and can even threaten their lives.^6^

Stroke is the world’s second greatest cause of disability and death, with low- and middle-income countries bearing the majority of the illness burden.^7^^,^^8^ In 2016, the number of incident new strokes increased to 13.7 million.^8^ In the same year, stroke caused 5.5 million deaths worldwide, with ischemic stroke accounting for 2.7 million and hemorrhagic stroke accounting for 2.8 million deaths, respectively.^8^ In 2016, the global stroke prevalence was 80.1 million [95% confidence intervals (CIs): 74.1–86.3], with 41.1 million (95% CI: 38.0–44.3) women and 39.0 million (95% CI: 36.1–42.1) males.^8^ Stroke is the second biggest cause of disability, accounting for approximately 116 million global disability-adjusted life-years lost in 2016.^8^ Lacunar stroke is a particular form of stroke characterized by a subcortical infarct measuring less than 20 mm in diameter, caused by occlusion of a perforator of an intracranial artery.^9^ The incidence of lacunar stroke varies based on the population studied from 25 to 50 per 100 000 people, accounting for 15%–25% of ischemic stroke.^9^^,^^10^^,^^11^^,^^12^ Globally, in 2019, the value of lost welfare due to stroke was $2059.67 billion or 1.66% of the global gross domestic product.^13^ As a result, understanding the causes and risk factors of stroke is crucial for early prevention and therapy, lowering stroke risk, and increasing overall quality of life. A population-based cohort study reported that PBC cohorts had stroke incidence rates of 13.8 (10.5–18.2) per 1,000 person-years. Hazard ratios in PBC cohort compared with the control cohort were for stroke 0.98 (95% CI: 0.73–1.31).^14^ In this cohort, PBC was not linked to a higher risk of stroke.^14^ Another study found that the risk of stroke was similar in the PBC and HCV infection groups.^15^ Asymptomatic PBC individuals are not more likely to develop cardiovascular disease.^15^^,^^16^ Given the ongoing debate, this study aims to elucidate the causality underlying the potential link between PBC and lacunar stroke.

Mendelian randomization (MR) studies, when combined with genome-wide association study (GWAS) summary statistics, have emerged as a potent, effective, and efficient way to determine the causal links between exposure phenotypes and outcomes.^17^^,^^18^ Genetic variations in GWAS data mimic randomized controlled trials by acting as fixed pre-birth proxies for risk factors in observational studies.^19^^,^^20^ MR studies have been widely used in recent years to investigate potential causal associations between various exposure elements and clinical outcomes. However, no study has applied the MR method to assess the correlation between PBC and lacunar stroke.

Therefore, this study used a two-sample MR approach to investigate the potential causal relationship between PBC and lacunar strokes. In this study, we used single nucleotide polymorphisms (SNPs) strongly associated with PBC as instrumental variables (IVs). Two-sample MR analysis greatly increases the scope and statistical power of MR using the published summary data from the GWAS database.^21^^,^^22^^,^^23^ The aim of this study was to assess whether PBC causally contributes to an increased risk of lacunar stroke.

## Results

### Preliminary identification of associations

#### Determination of instrumental variables and determination of bias in weak instrumental variables

In the initial phase of our MR analysis, we identified the IVs to explore the relationship between PBC and stroke (Table 1). After excluding SNPs with sequential imbalances from the GWAS data, 22 SNPs related to PBC were selected as IVs, meeting the stringent criteria of R^2^ < 0.001 and *p* < 5 × 10^−8^ (Table 2). These retained SNPs all exhibited F-statistics above 10, indicating a minimal impact of weak instrument bias and validating the reliability of our findings. Advancing our exploration, we narrowed our focus to the relationship between PBC and lacunar stroke using MR analysis. Following the exclusion of imbalanced IVs, 22 SNPs implicated in PBC were selected from the GWAS (R^2^ < 0.001, *p* < 5 × 10^−8^), and only those with F-statistics exceeding 10 were included to mitigate potential bias (Table 2).

**Table 1:** Summary of the GWAS included in this study

	Trait ID in GWAS	Sample size	Number of SNPs	Population	Sex	References	Year	Author
PBC	ebi-a-GCST005581	11375	119756	European	–	Liu et al.^21^	2012	Liu JZ
Stroke	ebi-a-GCST90038613	484598	9587836	European	–	Dönertaş et al.^22^	2021	Handan Melike Dönertaş
Lacunar stroke	ebi-a-GCST90014122	225419	6909434	European	–	Traylor et al.^23^	2021	Traylor M
PBC	ebi-a-GCST003129	13239	1124241	European	–	Cordell et al.^24^	2015	Cordell HJ
PBC	ebi-a-GCST90061440	24510	5004018	European	–	Cordell et al.^25^	2021	Cordell HJ

GWAS, genome-wide association study; PBC, primary biliary cholangitis; SNP, single-nucleotide polymorphism.

**Table 2:** PBC genetic IVs.Beta: the regression coefficient based on PBC raising effect allele

Exposure	SNP	Effect_allele	Other_allele	Beta	SE	*p* value	F	chr.exposure
PBC	rs10931468	A	C	0.319907	0.0423732	4.36E-14	56.99870129	2
PBC	rs11065979	T	C	0.183155	0.0308406	2.87E-09	35.26890051	12
PBC	rs11117431	G	A	−0.23471	0.0409413	9.88E-09	32.86547623	16
PBC	rs12134279	T	C	0.248421	0.0358054	3.97E-12	48.13705887	1
PBC	rs12708716	G	A	−0.236736	0.0330557	7.97E-13	51.2903773	16
PBC	rs1646019	T	C	−0.274305	0.0354	9.28E-15	60.04279823	16
PBC	rs17122453	A	G	−0.326839	0.0405574	7.71E-16	64.94228033	11
PBC	rs1800693	C	T	0.24059	0.0311702	1.18E-14	59.57663455	12
PBC	rs2267407	A	G	0.259283	0.0350056	1.29E-13	54.86217665	22
PBC	rs2293370	A	G	−0.337293	0.0417781	6.83E-16	65.18043975	3
PBC	rs2523882	T	C	0.227136	0.0337129	1.61E-11	45.39204217	6
PBC	rs3135024	C	T	0.506818	0.0338025	8.10E-51	224.8052341	6
PBC	rs34725611	G	A	−0.204935	0.0348862	4.24E-09	34.50840924	19
PBC	rs35188261	A	G	0.416735	0.0433154	6.52E-22	92.56254669	7
PBC	rs522127	C	A	−0.308974	0.0318494	2.98E-22	94.11121064	3
PBC	rs6871748	C	T	−0.263705	0.035965	2.26E-13	53.76214643	5
PBC	rs72678531	C	T	0.479954	0.0370728	2.47E-38	167.6055727	1
PBC	rs7665090	G	A	0.229162	0.0307076	8.48E-14	55.69208254	4
PBC	rs7775055	C	T	1.11317	0.0749024	5.85E-50	220.8673499	6
PBC	rs79513546	G	A	−1.52005	0.212086	7.66E-13	51.36789213	1
PBC	rs8067378	G	A	0.232058	0.0309124	6.05E-14	56.35437544	17
PBC	rs911263	T	C	0.227654	0.0351986	9.95E-11	41.83114872	14

SE, standard error; PBC, primary biliary cholangitis; IVs, instrumental variants; SNP, single-nucleotide polymorphism.

#### The effect of PBC on the risk of developing lacunar stroke

**Figure 1:**
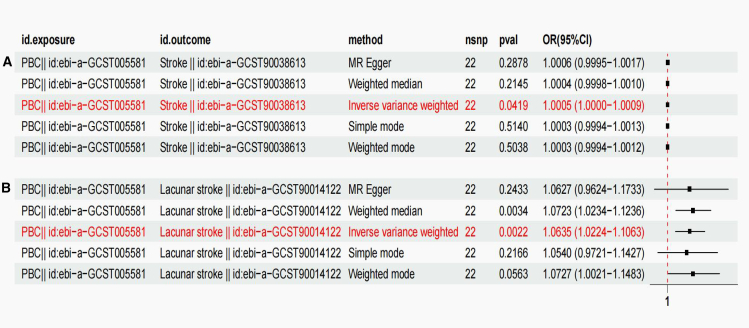
Forest plot of genetic association models Four methods were used to visualize the causal effect of PBC on stroke (A) and lacunar stroke (B) outcomes.

The effects of these SNPs on stroke (Figure S1A) and lacunar stroke were elucidated by MR analysis (Figure S1B). Figure S1C depicts evidence from genetic prediction, suggesting a strong link between PBC and stroke occurrence, with an OR of 1.0005 (95% CI: 1.0000–1.0009; *p* = 0.0419). Comparable outcomes were not observed when employing alternative methods, such as weighted mode, weighted median, and MR-Egger regression analyses (Figure 1). To further validate our findings, supplementary analyses using MR-Egger and MR-PRESSO were conducted on the implicated SNP loci. No horizontal pleiotropy was observed in these assessments (*p* > 0.05). A funnel plot confirmed the absence of bias in the study (Figure S2A).

Additionally, we discovered that PBC was strongly correlated with lacunar stroke according to the genetic prediction data, with an OR of 1.0635 (95% CI: 1.0224–1.1063; *p* = 0.0012) (Figure S1D). Similar causality was shown by weighted median regression (OR = 1.0732, 95% CI: 1.0234–1.1236, *p* = 0.0034). No similar results were found in the MR-Egger regression analysis (Figure 1). To confirm the stability of the above results, MR-Egger and MR-PRESSO analyses were conducted on the included SNP sites, and no potential horizontal pleiotropy was detected (*p* > 0.05), which further demonstrated the lack of bias in this investigation (Figure S2B).

#### Sensitivity analysis

Leave-one-out sensitivity analysis was employed to scrutinize the contribution of each SNP to the overall causality, with each SNP being systematically excluded from the analysis. The MR analyses, as detailed in Figures S2C and S2D, demonstrated that the causal patterns remained stable after exclusion, suggesting that the aggregate effect is not attributable to any single genetic variant. Notably, the IVW method, which is less susceptible to the influence of heterogeneity, corroborated our findings and reinforced the reliability of our results.

To further assess the directionality of the causal relationship, we performed MR Steiger tests to rule out reverse causation. The results showed that the genetic instruments explained significantly more variance in PBC (R^2^ = 14.6%) than in lacunar stroke (R^2^ = 0.0002%, Steiger *p* < 2.2 × 10^−16^), with the correct_causal_direction confirmed as TRUE. This provides strong evidence that the observed association reflects a causal effect of PBC on lacunar stroke, rather than reverse causation (Table S1).

### Sensitivity analysis in an updated PBC GWAS

#### Expanded evidence: Investigating the PBC-lacunar stroke link

**Figure 2:**
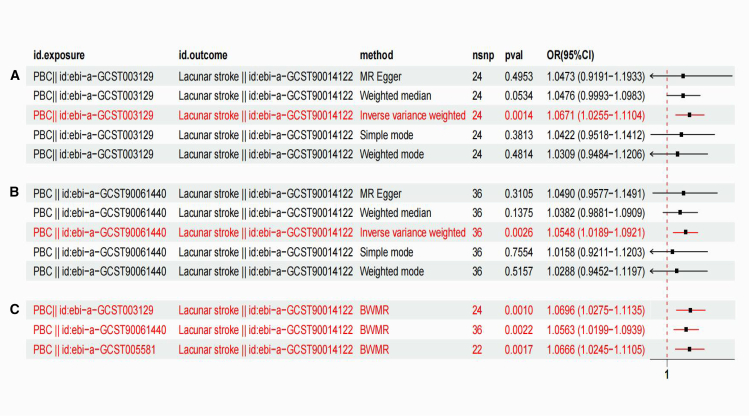
Genetic associations between PBC and lacunar stroke in an updated GWAS and BWMR sensitivity analysis (A and B) Forest plots of genetic associations between PBC and lacunar stroke from an updated PBC GWAS. (C) Forest plot from BWMR sensitivity analysis.

To evaluate the causal link between PBC and lacunar stroke, we assessed the causal link between PBC and lacunar stroke using 24 SNPs that were significantly associated with PBC (*p* < 5 × 10^−8^), all with F-statistics >10, ensuring reliable results. IVW method analysis showed an OR of 1.0671 (95% CI: 1.0255–1.110; *p* = 0.0014), indicating a significant association (Figure 2A). Horizontal pleiotropy was not detected by MR-Egger regression or MR-PRESSO testing (*p* > 0.05), and funnel plot analysis revealed no asymmetry, confirming the robustness of the results. A leave-one-out analysis confirmed the stability of our causal estimate, independent of the individual SNPs (Figure S3).

#### Stability in the latest meta-analysis: Confirming the PBC-Lacunar stroke association

To further corroborate the robustness of the causal estimate, 37 SNPs significantly associated with PBC were used as IVs to assess their impact on lacunar stroke. All SNPs had F-statistics greater than 10, indicating reliable results without bias from instrument variable effects. Analysis using the IVW method revealed an OR of 1.0548 (95% CI: 1.0189–1.0921; *p* = 0.0026), suggesting a significant positive association between PBC and lacunar stroke (Figure 2B**)**. Neither MR-Egger regression nor MR-PRESSO testing found evidence of horizontal pleiotropy (*p* > 0.05), and funnel plot analysis did not show significant asymmetry, further supporting the robustness of the results. Furthermore, we validated the robustness of our results using leave-one-out SNP analysis, confirming that the exclusion of individual SNPs did not alter the overall causal estimates (Figure S4).

#### BWMR analysis

To further enhance the robustness and reliability of our findings, we expanded upon our initial IVW analysis by incorporating BWMR. This approach assigns weights to each genetic instrument, effectively reducing potential pleiotropy and integrating multiple evidence sources. Consequently, BWMR provides a more detailed assessment of uncertainty and sensitivity, deepening our understanding of the causal relationship between PBC and lacunar stroke. The BWMR analysis revealed significant associations, with *p* values of 0.0017, 0.0010, and 0.0022 for the respective phases of PBC data (Figure 2C**)**. By integrating BWMR into our methodology, we not only strengthened the credibility of our results but also underscored the complementary strengths of different statistical techniques. This integrative strategy offers a solid foundation for investigating complex causal relationships.

## Discussion

In this study, we employed a two-sample MR approach to investigate the causal link between PBC and lacunar strokes. Our analysis was meticulously designed in two phases: an initial identification phase followed by an external validation phase utilizing two independent PBC datasets to ensure the robustness of our findings. Our results revealed that PBC is a significant risk factor for lacunar stroke. These discoveries are consistent with those of other studies that have employed diverse methodologies, thereby corroborating the strong association between PBC and lacunar stroke. Our research underscores the marked escalation in lacunar stroke risk associated with PBC progression.

While our MR analysis supports a causal role of PBC in increasing the risk of lacunar stroke, this finding contrasts with prior observational evidence, which has not consistently demonstrated such an association. A UK-based cohort study of 930 patients with PBC reported no significant increase in the risk of lacunar stroke or transient ischemic attack, a null result that may reflect methodological limitations inherent to observational designs.^14^ These include insufficient statistical power due to the low incidence of lacunar infarction, as well as potential misclassification of stroke subtypes—lacunar infarcts are frequently diagnosed based on clinical criteria or non-specific imaging in routine practice, and the absence of centralized neuroimaging adjudication may lead to non-differential misclassification, biasing associations toward the null.^26^ Residual confounding is another critical concern, particularly from treatment effects such as immunosuppressive therapy and, more notably, the widespread use of ursodeoxycholic acid (UDCA), which has been associated with reduced systemic inflammation and improved vascular function in PBC.^27^ This therapeutic benefit may attenuate cerebrovascular risk in clinical cohorts, thereby obscuring underlying pathological associations. In contrast, our two-sample MR approach leverages genetic variants as IVs for PBC, modeling lifelong exposure to the disease trait while minimizing the influence of time-varying confounders, treatment effects, and reverse causation. This design offers greater sensitivity for detecting causal effects, particularly for outcomes with long latency or low incidence. Notably, the same observational cohort identified a significantly elevated risk of subarachnoid hemorrhage among PBC patients (rate ratio = 2.21, 95% CI: 1.43–3.16), suggesting that cerebrovascular vulnerability is indeed present in this population.^28^ Given that both subarachnoid hemorrhage and lacunar stroke share underlying pathological features—particularly cerebral small vessel disease—this finding supports the plausibility of a systemic vasculopathic process in PBC, warranting further investigation into its biological underpinnings.

This growing epidemiological evidence aligns with emerging insights into the systemic manifestations of PBC beyond the hepatobiliary system. Chronic immune activation in PBC is characterized by sustained elevation of pro-inflammatory cytokines, including interleukin-6 and tumor necrosis factor-alpha, which promote endothelial dysfunction by reducing nitric oxide bioavailability and increasing vascular permeability—key initiating events in cerebral small vessel disease.^29^ Autoimmune-mediated vascular injury, previously documented in primary sclerosing cholangitis as a mechanism of bile duct ischemia, may similarly affect cerebral perforating arteries, which are particularly vulnerable in lacunar infarction. Supporting this hypothesis, elevated circulating levels of intercellular adhesion molecule 3 (ICAM-3), a biomarker of endothelial activation and leukocyte-endothelial interaction, have been prospectively associated with increased ischemic stroke risk in population-based cohorts.^30^^,^^31^ Furthermore, dyslipidemia in PBC—marked by elevated low-density lipoprotein cholesterol and total cholesterol, alongside functionally impaired high-density lipoprotein—contributes to a pro-atherogenic milieu.^32^ Lipidomic analyses reveal dysregulation of glycerophospholipid and sphingolipid metabolism in PBC, pathways directly implicated in endothelial dysfunction and atherosclerosis.^33^ Disrupted enterohepatic bile acid circulation further exacerbates systemic metabolic disturbances that may accelerate cerebrovascular disease.^34^^,^^35^ Critically, a recent bidirectional multivariable MR study by Liu et al. provided independent genetic evidence that PBC causally increases the risk of small-vessel ischemic stroke, reinforcing the biological plausibility of our findings.^36^ When combined with chronic inflammation, this pro-atherogenic profile may accelerate atherosclerosis in cerebral small arteries. Indeed, long-term data from the women’s health study demonstrate that midlife LDL elevation strongly predicts stroke risk over three decades, particularly in inflammatory contexts.^37^ Together, these lines of evidence suggest that the convergence of immune dysregulation, endothelial injury, and metabolic disturbance in PBC creates a multifactorial substrate for lacunar stroke.

This study has several key strengths. First, we adopted a two-stage analytical framework, beginning with a discovery phase followed by sensitivity analyses across multiple temporally progressive and increasingly large-scale GWASs of PBC and lacunar stroke. Although some degree of sample overlap may exist—particularly between earlier PBC GWASs—we note that the most recent datasets incorporate substantial new contributions from international consortia, including the Canadian, Chinese, Italian, Japanese, US, and UK PBC Consortia. This expansion in sample diversity and size supports a more robust assessment of result consistency over time, thereby enhancing confidence in the reproducibility of our findings. Second, all genetic instruments were selected based on stringent criteria, including genome-wide significance (*p* < 5 × 10^−8^) and strong instrument strength (F-statistic >10), minimizing the risk of weak instrument bias and improving the reliability of causal estimates. Third, the exposure data were derived from large-scale, well-powered GWASs of European ancestry, ensuring high precision in effect estimation. While the outcome datasets include participants from European, North American, and Australasian cohorts, the predominant European ancestry aligns with the exposure data, reducing concerns about population stratification and supporting genetic comparability. Finally, we applied BWMR, a state-of-the-art MR method that provides consistent causal estimates even when up to 50% of the genetic instruments are invalid due to horizontal pleiotropy. By downweighting or excluding outliers, BWMR increases robustness against pleiotropic bias and strengthens the validity of our causal inferences. Together, these methodological features support a rigorous and transparent evaluation of the potential causal relationship between PBC and lacunar stroke.

### Conclusion

In conclusion, the current study established a causal link between PBC and the risk of lacunar stroke from a genetic perspective. Individuals with PBC have an elevated risk of lacunar strokes. It is essential to implement early screening for lacunar stroke in patients with PBC to enable preemptive prevention and intervention. Therefore, we recommend regular lacunar stroke risk assessment for individuals with PBC. Further research is required to clarify the mechanisms underlying this relationship.

### Limitations of the study

Nonetheless, several limitations warrant consideration. First, our analysis relied on summary-level GWAS data, which precluded adjustment for important clinical covariates, such as immunosuppressive therapy, disease duration, liver function parameters, and systemic inflammatory markers. These factors may lie on the causal pathway between PBC and cerebrovascular outcomes, and their absence limits our ability to assess mediation or time-varying effects. Although MR is inherently robust to conventional confounding, unmeasured biological pathways—particularly chronic inflammation or shared autoimmune mechanisms—could still violate the exclusion restriction assumption if they independently influence both the genetic instruments and the outcome. Second, the lack of individual-level data prevented stratified or subgroup analyses by age, sex, or disease severity, restricting our capacity to explore heterogeneity in the causal effect across different populations. Third, while sensitivity analyses—including MR-Egger intercept, Cochran’s Q, and MR-PRESSO—showed no significant evidence of directional pleiotropy or outlier bias, residual confounding cannot be entirely ruled out. Furthermore, due to the limited availability of large-scale PBC GWASs, our sensitivity analyses included all currently accessible datasets; however, true external validation in fully independent, ancestrally diverse, or prospectively phenotyped cohorts remains an important goal for future research.

## Resource availability

### Lead contact

Further information and requests for resources should be directed to and will be fulfilled by the lead contact, Haichu Yu (haichuyu@163.com).

### Materials availability

This study did not generate new unique reagents or biological materials.

### Data and code availability

• Data: GWAS summary statistics analyzed in this study are publicly available from the IEU OpenGWAS database (https://gwas.mrcieu.ac.uk/) under the study identifiers listed in the key resources table: Primary biliary cholangitis (PBC): GCST005581, GCST003129, GCST90061440;Lacunar stroke: GCST90014122;Ischemic stroke: GCST90038613
• Code: The complete R code for Mendelian randomization analyses, sensitivity tests, and figure generation is provided as Data S1: Supplementary_Code_S1_MR_Analysis.pdf (see “Other” entry in the key resources table).
• Other: All software tools used are publicly available and documented in the key resources table, including R (RRID:SCR_001905), the TwoSampleMR package (RRID:SCR_018468), MR-PRESSO, and GWAS Atlas.

## Acknowledgments

The authors extend their gratitude to all researchers who generously shared GWAS summary data that supported this study. The 10.13039/100014718National Natural Science Foundation of China (grant no. 82200401) and the 10.13039/501100010870Qingdao Municipal Science and Technology Bureau (award no. 20-3-4-54-nsh).

## Author contributions

M.W.: Conceptualization, formal Analysis, data curation, visualization, writing − original draft, writing − review & editing. Nan Zhang: formal analysis, data curation, writing-original draft. L.Z.: formal analysis, data curation, writing-original draft. Ning Zhang: conceptualization, methodology, supervision, writing − review & editing. H.Y.: conceptualization, funding acquisition, supervision, writing − review & editing. All authors: project administration, final approval of manuscript.

## Declaration of interests

The authors declare no competing interests.

## STAR★Methods

### Key resources table

**Table:** 

REAGENT or RESOURCE	SOURCE	IDENTIFIER
**Deposited Data**		

GWAS summary statistics for PBC, lacunar stroke, and ischemic stroke	IEU Open GWAS project	https://gwas.mrcieu.ac.uk
Individual-level GWAS data for PBC (study IDs: GCST005581, GCST003129, GCST90061440)	IEU Open GWAS project	https://gwas.mrcieu.ac.uk
Individual-level GWAS data for stroke (GCST90038613) and lacunar stroke (GCST90014122)	IEU Open GWAS project	https://gwas.mrcieu.ac.uk

**Software and Algorithms**		

R statistical software	R Foundation for Statistical Computing	RRID: SCR_001905; https://www.r-project.org
TwoSampleMR R package	Hemani et al.^38^	RRID: SCR_018468; https://mrcieu.github.io/TwoSampleMR/
MR-PRESSO R package	Verbanck et al.^39^	https://github.com/rondolab/MR-PRESSO
GWAS Atlas	GWAS Atlas Consortium	https://gwasatlas.org
Adobe Illustrator	Adobe Systems	N/A

**Other**		

Analysis code (R scripts for MR analyses)	This paper	Data S1

### Experimental model and study participant details

This study was conducted in two sequential phases to systematically examine the potential association between PBC and lacunar stroke. In the first phase, we employed a two-sample MR approach using GWAS summary statistics to investigate the causal effect of genetically predicted PBC on lacunar stroke risk. To assess the robustness of our findings, we performed comprehensive sensitivity analyses using PBC-associated SNPs as IVs. Building on these results, the second phase aimed to evaluate the consistency of the observed association across updated and expanded PBC datasets, including large-scale meta-analyses with increased sample size and broader geographic representation. While some overlap in study populations cannot be ruled out, this approach allows for a more rigorous assessment of result stability over time. To account for potential horizontal pleiotropy and strengthen causal inference, we applied Bayesian weighted MR (BWMR), a robust method that downweights outlying instruments and provides reliable estimates even in the presence of invalid instruments. This two-phase strategy enhances confidence in the reproducibility and validity of the findings.

The primary datasets used in this research were as follows: the ebi-a-GCST005581 dataset for primary biliary cholangitis (PBC), which included 2,861 cases and 8,514 controls^21^; stroke data from the ebi-a-GCST90038613 dataset, involving 6,925 cases and 477,673 controls^22^; and lacunar stroke data from the ebi-a-GCST90014122 dataset, including 6,030 cases and 248,929 controls^23^ (Table 1).

In the second phase of our study, we extended the analysis to two additional European-ancestry PBC GWAS datasets to assess the consistency of our findings. The first dataset (ebi-a-GCST003129, 2015) included 2,764 cases and 10,475 controls.^24^ The second dataset (ebi-a-GCST90061440, 2021) comprised 8,021 cases and 16,489 controls^25^ (Table 1).

All analyses were based on publicly available summarized GWAS data. Ethical approval and participant consent had been obtained for the original studies from which the data were derived.

#### Instrumental variable selection

To investigate our initial hypothesis, we focused on SNPs demonstrating robust genome-wide significance in relation to PBC (P-value threshold: 5 × 10^−8^, linkage disequilibrium threshold: R^2^ < 0.001, and a clumping window size of 10,000 kb).^40^^,^^41^ Proceeding this, we consulted the GWAS Atlas (available at https://gwasatlas.org), to verify the absence of links between these genetic markers and any probable confounding factors, thereby reinforcing our secondary hypothesis.^42^ We then applied a heterogeneity test to filter out SNPs exhibiting significant inconsistency, retaining only those SNPs with a substantial association to PBC, which were subsequently designated as IVs.

To delve deeper into the association hypothesis, we meticulously examined the potential for bias introduced by weak instrumental variables by calculating the F-statistic. A threshold of F exceeding 10 was established as indicative of an acceptable lack of bias within our instrumental variable selection. This F-statistic, computed using the formula F, equals the squared effect size (β) divided by the squared standard error (SE),^43^ and serves as a crucial measure in assessing the robustness of our instrumental variable assumptions.

#### Two-sample mendelian randomization analysis

In this study, we performed comprehensive two-sample MR analyses utilizing techniques such as random-effects inverse variance weighting (IVW), the MR-Egger approach, weighted model, and weighted median method to assess the probable causal relationship between PBC and lacunar stroke, as expressed by odds ratio (OR) measurements. The standard application of the IVW method may encounter biases arising from unreliable instrumental variables or pleiotropy. Nonetheless, when homogeneous data and an absence of pleiotropic effects are assured, IVW is acknowledged to yield the most reliable and precise causal inferences.^44^ Consequently, this study included a sensitivity analysis to critically assess and confirm the robustness and credibility of the conclusions drawn from the IVW methodology.^45^

To rigorously address and alleviate any potential repercussions from horizontal pleiotropy, thereby enhancing the reliability of our findings, this research employed the MR-PRESSO approach.^46^ This method is instrumental in identifying and correcting for pervasive horizontal pleiotropy across all analyses. A key indicator suggesting the absence of such pleiotropy is when the intercept term approximates zero and the corresponding *p*-value exceeds 0.05, suggesting that any bias due to pleiotropic effects is minimal.^47^^,^^48^^,^^49^ Moreover, we employed the MR-Egger regression and a weighted median approach as supplementary means to examine the PBC-lacunar stroke association. To strengthen the reliability of our findings, we also conducted heterogeneity assessments of statistically significant outcomes, incorporating procedures such as the MR-Egger intercept examination, sensitivity evaluations, and utilization of a modified Cochran’s Q statistic.^47^^,^^50^

Similarly, we adopted a funnel plot technique, a method similar to the one popular in meta-analysis for revealing publication biases, to assess the potential for directional pleiotropy.^46^ Moreover, to guard against the confusion that can arise from incorrectly assuming cause and effect. We applied MR Steiger tests, enhancing the reliability of our findings.^51^^,^^52^ Furthermore, we employed the leave-one-out methodology in our sensitivity analyses. At each step, a single SNP was iteratively removed, the outcome prediction was recalculated with the remaining SNPs, and this new result was contrasted with the combined estimate, thereby enabling the individual impacts of each SNP to be discerned.

### Quantification and statistical analysis

All MR analyses were conducted using the ‘TwoSampleMR’ package in R (version 4.4.1), with additional statistical operations performed in EmpowerStats. The analytical framework followed a two-phase approach: an initial discovery analysis using a primary PBC GWAS dataset, followed by a replication analysis in two updated European-ancestry PBC datasets to assess the consistency of findings. MR analyses rely on three core IV assumptions^20^: (1) relevance—the IVs must be strongly associated with the exposure (PBC); (2) independence—the IVs should not be associated with confounders of the exposure–outcome relationship; and (3) exclusion restriction—the IVs influence the outcome (lacunar stroke) only through their effect on the exposure, with no direct or pleiotropic pathways. Results are reported as ORs with 95% CIs to quantify the causal effect of PBC on lacunar stroke risk. Statistical significance was defined as *p* < 0.05 (two-sided tests). Figures were generated using Adobe Illustrator 2021.

## References

[1] Levy C., Manns M., Hirschfield G. (2023). New Treatment Paradigms in Primary Biliary Cholangitis. Clin. Gastroenterol. Hepatol..

[2] Lindor K.D., Gershwin M.E., Poupon R., Kaplan M., Bergasa N.V., Heathcote E.J., American Association for Study of Liver Diseases (2009). Primary biliary cirrhosis. Hepatology.

[3] Frazer I.H., Mackay I.R., Jordan T.W., Whittingham S., Marzuki S. (1985). Reactivity of anti-mitochondrial autoantibodies in primary biliary cirrhosis: definition of two novel mitochondrial polypeptide autoantigens. J. Immunol..

[4] Talwalkar J.A., Lindor K.D. (2003). Primary biliary cirrhosis. Lancet (London, England).

[5] Boonstra K., Beuers U., Ponsioen C.Y. (2012). Epidemiology of primary sclerosing cholangitis and primary biliary cirrhosis: a systematic review. J. Hepatol..

[6] You H., Ma X., Efe C., Wang G., Jeong S.H., Abe K., Duan W., Chen S., Kong Y., Zhang D. (2022). APASL clinical practice guidance: the diagnosis and management of patients with primary biliary cholangitis. Hepatol. Int..

[7] Saini V., Guada L., Yavagal D.R. (2021). Global Epidemiology of Stroke and Access to Acute Ischemic Stroke Interventions. Neurology.

[8] GBD 2016 Stroke Collaborators (2019). Global, regional, and national burden of stroke, 1990-2016: a systematic analysis for the Global Burden of Disease Study 2016. Lancet Neurol..

[9] Wardlaw J.M., Smith E.E., Biessels G.J., Cordonnier C., Fazekas F., Frayne R., Lindley R.I., O'Brien J.T., Barkhof F., Benavente O.R. (2013). Neuroimaging standards for research into small vessel disease and its contribution to ageing and neurodegeneration. Lancet Neurol..

[10] Sacco S., Marini C., Totaro R., Russo T., Cerone D., Carolei A. (2006). A population-based study of the incidence and prognosis of lacunar stroke. Neurology.

[11] Yaghi S., Raz E., Yang D., Cutting S., Mac G.B., Elkind M.S., de Havenon A. (2021). Lacunar stroke: mechanisms and therapeutic implications. J. Neurol. Neuro. Psych..

[12] Petty G.W., Brown R.D., Whisnant J.P., Sicks J.D., O'Fallon W.M., Wiebers D.O. (1999). Ischemic stroke subtypes: a population-based study of incidence and risk factors. Stroke.

[13] Gerstl J.V.E., Blitz S.E., Qu Q.R., Yearley A.G., Lassarén P., Lindberg R., Gupta S., Kappel A.D., Vicenty-Padilla J.C., Gaude E. (2023). Global, Regional, and National Economic Consequences of Stroke. Stroke.

[14] Solaymani-Dodaran M., Aithal G.P., Card T., West J. (2008). Risk of cardiovascular and cerebrovascular events in primary biliary cirrhosis: a population-based cohort study. Am. J. Gastroenterol..

[15] Doycheva I., Chen C., Pan J.J., Levy C. (2011). Asymptomatic primary biliary cirrhosis is not associated with increased frequency of cardiovascular disease. World J. Hepatol..

[16] Zalewski P., Jones D., Lewis I., Frith J., Newton J.L. (2013). Reduced thoracic fluid content in early-stage primary biliary cirrhosis that associates with impaired cardiac inotropy. Am. J. Physiol. Gastrointest. Liver Physiol..

[17] Burgess S., Dudbridge F., Thompson S.G. (2016). Combining information on multiple instrumental variables in Mendelian randomization: comparison of allele score and summarized data methods. Stat. Med..

[18] Wang M., Mo D., Zhou C., Zhang W., Chen R., Xu J., Zhang N., Yu H. (2024). Causal association between Neuroticism and risk of aortic aneurysm: A bidirectional two-sample Mendelian randomization study. J. Affect. Disord..

[19] Davey Smith G., Ebrahim S. (2005). What can mendelian randomisation tell us about modifiable behavioural and environmental exposures?. BMJ (Clinical research ed.).

[20] Davies N.M., Holmes M.V., Davey Smith G. (2018). Reading Mendelian randomisation studies: a guide, glossary, and checklist for clinicians. BMJ (Clinical research ed.).

[21] Liu J.Z., Almarri M.A., Gaffney D.J., Mells G.F., Jostins L., Cordell H.J., Ducker S.J., Day D.B., Heneghan M.A., Neuberger J.M. (2012). Dense fine-mapping study identifies new susceptibility loci for primary biliary cirrhosis. Nat. Genet..

[22] Dönertaş H.M., Fabian D.K., Fuentealba M., Partridge L., Thornton J.M. (2021). Common genetic associations between age-related diseases. Nat. Aging.

[23] Traylor M., Persyn E., Tomppo L., Klasson S., Abedi V., Bakker M.K., Torres N., Li L., Bell S., Rutten-Jacobs L. (2021). Genetic basis of lacunar stroke: a pooled analysis of individual patient data and genome-wide association studies. Lancet Neurol..

[24] Cordell H.J., Han Y., Mells G.F., Li Y., Hirschfield G.M., Greene C.S., Xie G., Juran B.D., Zhu D., Qian D.C. (2015). International genome-wide meta-analysis identifies new primary biliary cirrhosis risk loci and targetable pathogenic pathways. Nat. Commun..

[25] Cordell H.J., Fryett J.J., Ueno K., Darlay R., Aiba Y., Hitomi Y., Kawashima M., Nishida N., Khor S.S., Gervais O. (2021). An international genome-wide meta-analysis of primary biliary cholangitis: Novel risk loci and candidate drugs. J. Hepatol..

[26] Grover V.P.B., Southern L., Dyson J.K., Kim J.U., Crossey M.M.E., Wylezinska-Arridge M., Patel N., Fitzpatrick J.A., Bak-Bol A., Waldman A.D. (2016). Early primary biliary cholangitis is characterised by brain abnormalities on cerebral magnetic resonance imaging. Aliment. Pharmacol. Ther..

[27] van Hooff M.C., Werner E., van der Meer A.J. (2024). Treatment in primary biliary cholangitis: Beyond ursodeoxycholic acid. Eur. J. Intern. Med..

[28] Ramagopalan S.V., Pakpoor J., Seminog O., Goldacre R., Graham L., Goldacre M.J. (2013). Risk of subarachnoid haemorrhage in people admitted to hospital with selected immune-mediated diseases: record-linkage studies. BMC Neurol..

[29] Shi T.Y., Zhang F.C. (2012). Role of autoimmunity in primary biliary cirrhosis. World J. Gastroenterol..

[30] Pletsch-Borba L., Grafetstätter M., Hüsing A., Johnson T., González Maldonado S., Groß M.L., Kloss M., Hoffmeister M., Bugert P., Kaaks R., Kühn T. (2020). Vascular injury biomarkers and stroke risk: A population-based study. Neurology.

[31] Pollheimer M.J., Halilbasic E., Fickert P., Trauner M. (2011). Pathogenesis of primary sclerosing cholangitis. Best Pract. Res. Clin. Gastroenterol..

[32] Allocca M., Crosignani A., Gritti A., Ghilardi G., Gobatti D., Caruso D., Zuin M., Podda M., Battezzati P.M. (2006). Hypercholesterolaemia is not associated with early atherosclerotic lesions in primary biliary cirrhosis. Gut.

[33] Zheng L., Tian S., Yang C., Li B., Jia G., Liu Y., Sun R., Wang X., Deng J., Zhang M. (2024). Hypercholesterolemia Is Associated With Dysregulation of Lipid Metabolism and Poor Prognosis in Primary Biliary Cholangitis. Clin. Gastroenterol. Hepatol..

[34] Reshetnyak V.I. (2015). Primary biliary cirrhosis: Clinical and laboratory criteria for its diagnosis. World J. Gastroenterol..

[35] Wang H.H., Garruti G., Liu M., Portincasa P., Wang D.Q.H. (2017). Cholesterol and Lipoprotein Metabolism and Atherosclerosis: Recent Advances In reverse Cholesterol Transport. Ann. Hepatol..

[36] Lin Y.L., Yao T., Wang Y.W., Yu J.S., Zhen C., Lin J.F., Chen S.B. (2024). Association between primary biliary cholangitis with diabetes and cardiovascular diseases: A bidirectional multivariable Mendelian randomization study. Clin. Res. Hepatol. Gastroenterol..

[37] Ridker P.M., Moorthy M.V., Cook N.R., Rifai N., Lee I.M., Buring J.E. (2024). Inflammation, Cholesterol, Lipoprotein(a), and 30-Year Cardiovascular Outcomes in Women. N. Engl. J. Med..

[38] Hemani G., Zheng J., Elsworth B., Wade K.H., Baird D., Haberland V., Laurin C., Burgess S., Bowden J., Langdon R. (2018). The MR-Base platform supports systematic causal inference across the human phenome. eLife.

[39] Verbanck M., Chen C.Y., Neale B., Do R. (2018). Detection of widespread horizontal pleiotropy in causal relationships inferred from Mendelian randomization between complex traits and diseases. Nat. Genet..

[40] Quintana R.A., Taylor W.R. (2019). Introduction to the Compendium on Aortic Aneurysms. Circ. Res..

[41] Pritchard J.K., Przeworski M. (2001). Linkage disequilibrium in humans: models and data. Am. J. Hum. Genet..

[42] Kamat M.A., Blackshaw J.A., Young R., Surendran P., Burgess S., Danesh J., Butterworth A.S., Staley J.R. (2019). PhenoScanner V2: an expanded tool for searching human genotype-phenotype associations. Bioinformatics (Oxford, England).

[43] Burgess S., Thompson S.G., CRP CHD Genetics Collaboration (2011). Avoiding bias from weak instruments in Mendelian randomization studies. Int. J. Epidemiol..

[44] Burgess S., Thompson S.G. (2017). Interpreting findings from Mendelian randomization using the MR-Egger method. Eur. J. Epidemiol..

[45] Khankari N.K., Shu X.O., Wen W., Kraft P., Lindström S., Peters U., Schildkraut J., Schumacher F., Bofetta P., Risch A. (2016). Association between Adult Height and Risk of Colorectal, Lung, and Prostate Cancer: Results from Meta-analyses of Prospective Studies and Mendelian Randomization Analyses. PLoS Med..

[46] Bowden J., Davey Smith G., Burgess S. (2015). Mendelian randomization with invalid instruments: effect estimation and bias detection through Egger regression. Int. J. Epidemiol..

[47] Burgess S., Bowden J., Fall T., Ingelsson E., Thompson S.G. (2017). Sensitivity Analyses for Robust Causal Inference from Mendelian Randomization Analyses with Multiple Genetic Variants. Epidemiology.

[48] Hartwig F.P., Davey Smith G., Bowden J. (2017). Robust inference in summary data Mendelian randomization via the zero modal pleiotropy assumption. Int. J. Epidemiol..

[49] Verbanck M., Chen C.Y., Neale B., Do R. (2018). Detection of widespread horizontal pleiotropy in causal relationships inferred from Mendelian randomization between complex traits and diseases. Nat. Genet..

[50] Greco M F.D., Minelli C., Sheehan N.A., Thompson J.R. (2015). Detecting pleiotropy in Mendelian randomisation studies with summary data and a continuous outcome. Stat. Med..

[51] Davey Smith G., Hemani G. (2014). Mendelian randomization: genetic anchors for causal inference in epidemiological studies. Hum. Mol. Genet..

[52] Hemani G., Tilling K., Davey Smith G. (2017). Orienting the causal relationship between imprecisely measured traits using GWAS summary data. PLoS Genet..

