# Supplementary material for: Genetic evidence supports primary biliary cholangitis as a risk factor for lacunar stroke
Source: iScience. 2025 Nov 27;28(12):114240. doi: 10.1016/j.isci.2025.114240 (PMC12767174; doi:10.1016/j.isci.2025.114240)
Supplement: Document S1. Figures S1–S4, Table S1, and Data S1 [file mmc1.pdf]

iScience, Volume *28*

Supplemental information

Genetic evidence supports primary biliary cholangitis as a risk factor for lacunar stroke

Mengmeng Wang, Nan Zhang, Ling Zhang, Ning Zhang, and Haichu Yu

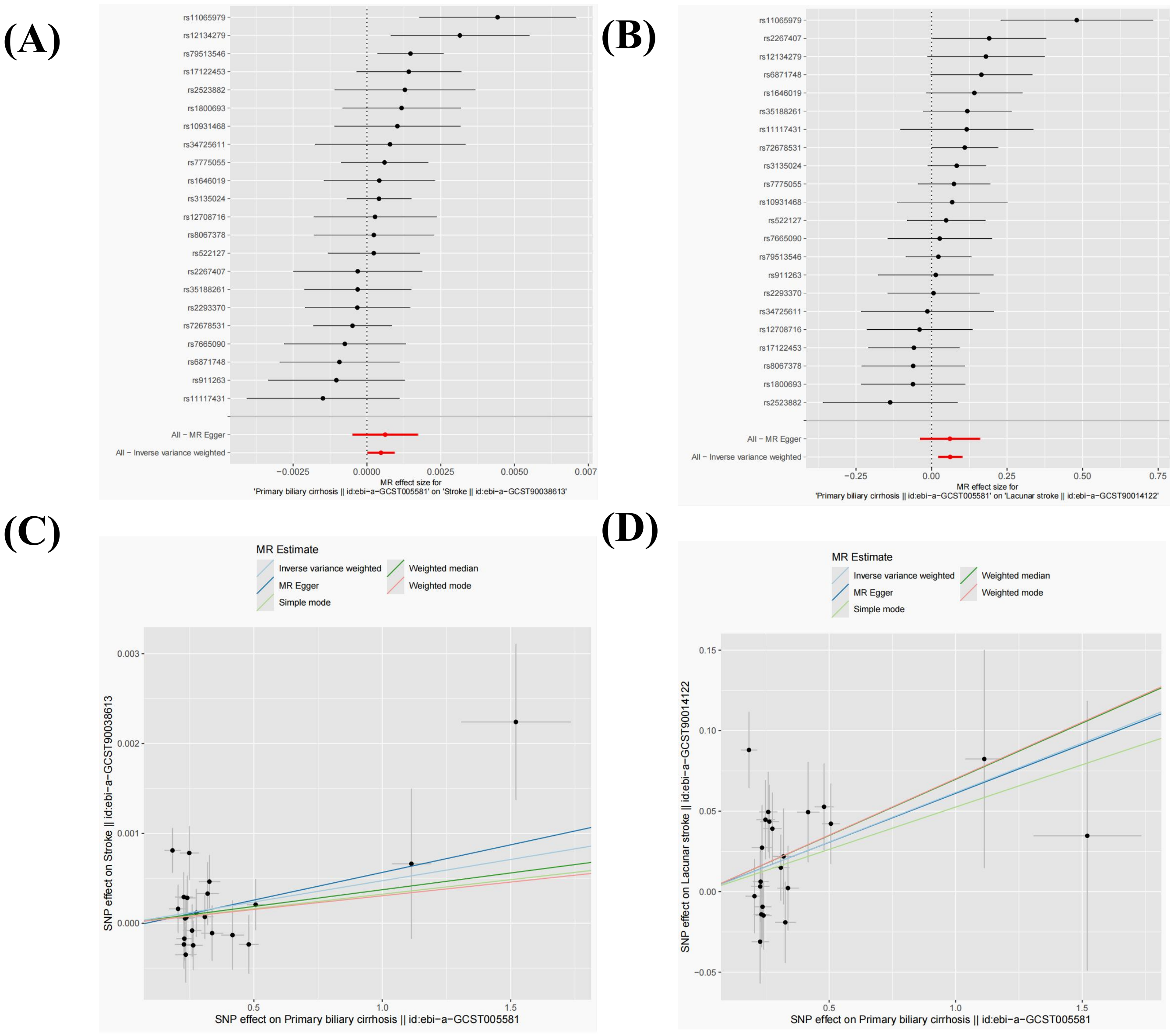

Supplementary Figure S1. Forest Plot and Scatter Plot for Causal Effects in PBC.

Forest plot to visualize causal effect of each single SNP of PBC and risk of stroke (A) and lacunar stroke (B). The scatter plot for the MR analyses of causal associations (C,D).

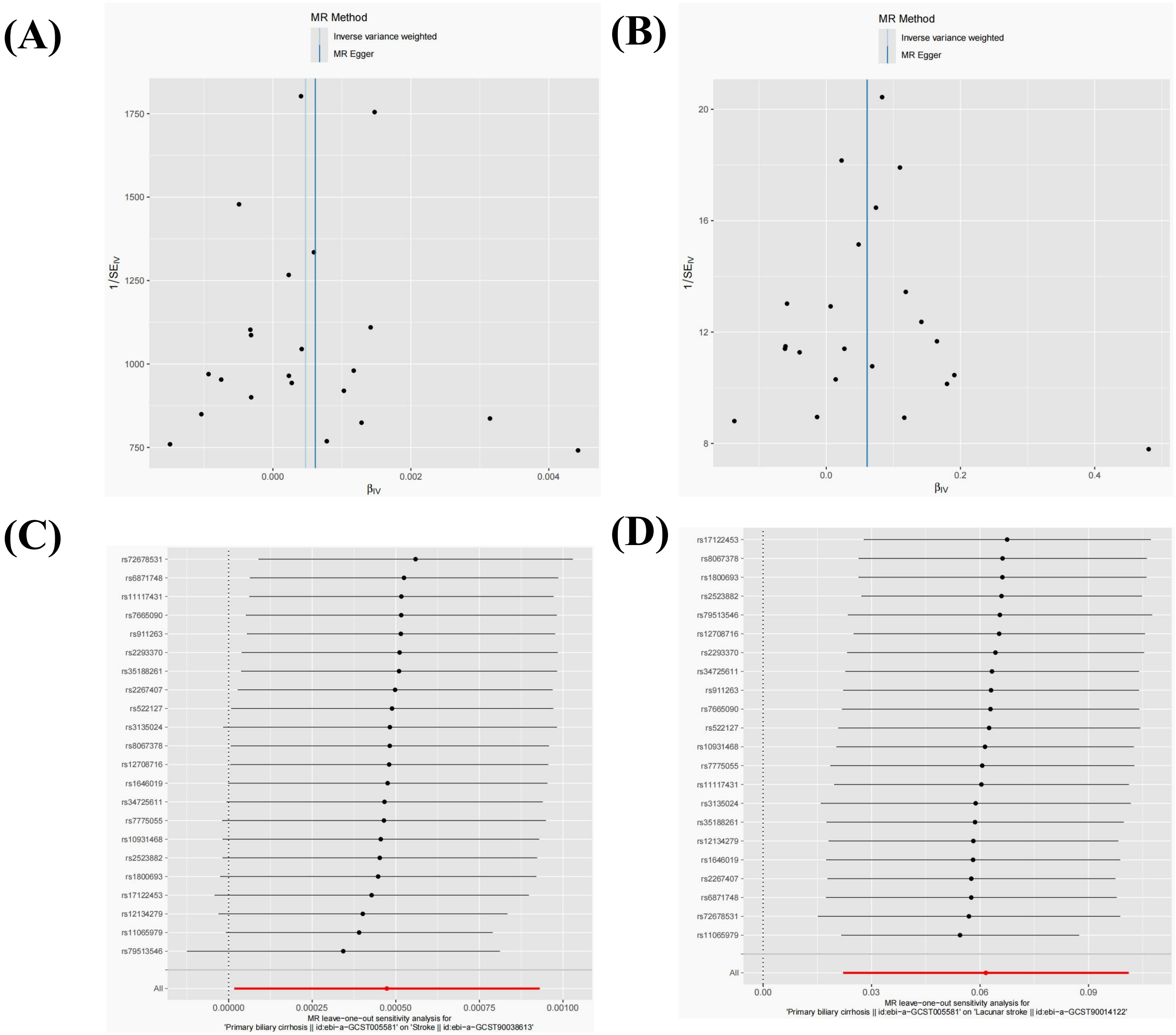

Supplementary Figure S2. Sensitivity and Validity Assessments in MR Analysis.
Funnel plot of the two-sample MR analysis (A, B). Leave-one-out sensitivity analysis for estimates of PBC on stroke (C) and lacunar stroke (D).

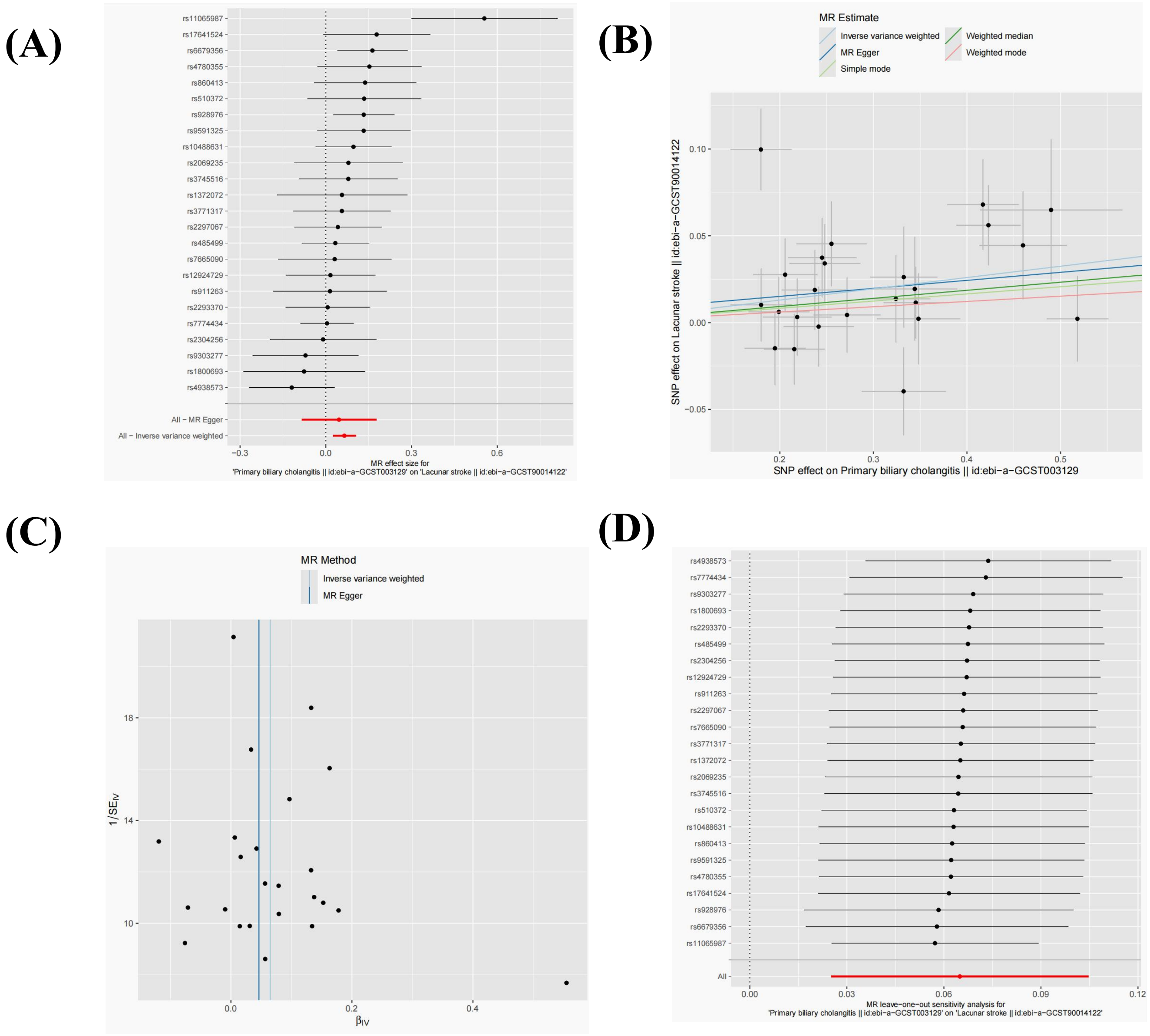

Supplementary Figure S3. Expanded MR Analysis. (A) Forest plot of SNP effects on lacunar stroke risk. (B) Scatter plot for MR analyses. (C) Funnel plot for MR analysis. (D) Leave-one-out sensitivity analysis for lacunar stroke risk.

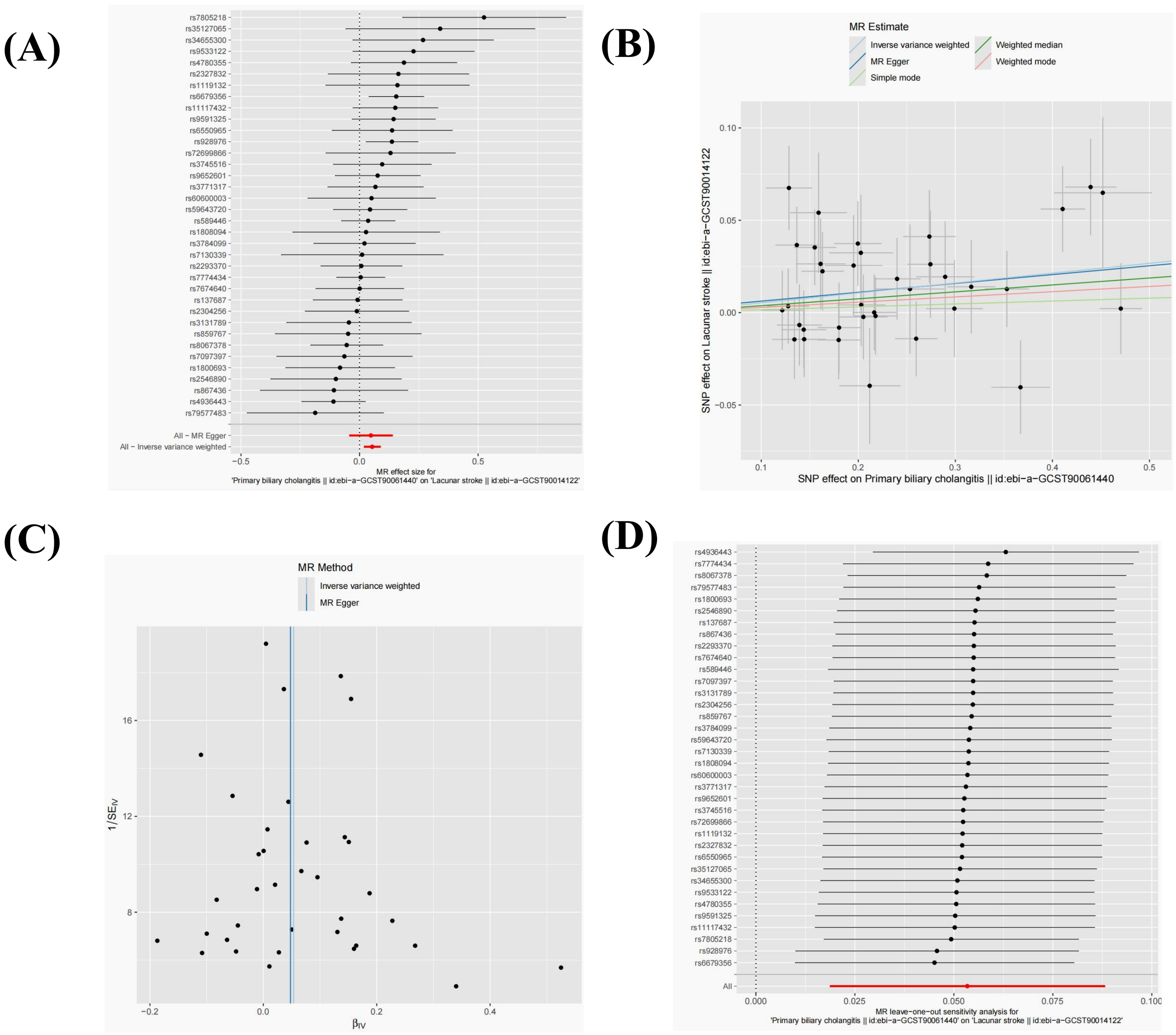

Supplementary Figure S4. Complementary MR Analysis of PBC and Lacunar Stroke. (A) Forest plot of SNP effects on lacunar stroke risk. (B) Scatter plot for MR analyses. (C) Funnel plot for MR analysis. (D) Leave-one-out sensitivity analysis for lacunar stroke risk.

Supplementary Table S1

mr_steiger_test.ebi-a-GCST005581.ebi-a-GCST90014122

id.exposure	id.outcome	exposure	outcome	snp_r2.exposure	snp_r2.outcome	correct_causal_direction	steiger_pval
ebi-a-GCST00558	bi-a-GCST9001412	Primary biliary cirrhosis ||	Lacunar stroke || id:ebi-a-GCST90014122	0.145993268	0.000175215	TRUE	0

mr_steiger_test.ebi-a-GCST005581.ebi-a-GCST90038613

id.exposure	id.outcome	exposure	outcome	snp_r2.exposure	snp_r2.outcome	correct_causal_direction	steiger_pval
ebi-a-GCST00558	bi-a-GCST9003861	Primary biliary cirrhosis ||	Stroke || id:ebi-a-GCST90038613	0.145993268	7.50E-05	TRUE	0

Supplementary_Code_S1_MR_Analysis

```
library(gtsummary)
library(survey)
library(haven)
library(tableone)
library(plyr)
library(dplyr)
library(tidyverse)
library(arsenal)
library(ggplot2)
library(TwoSampleMR)

library(TwoSampleMR)

exposure_data    <- extract_instruments(c('ebi-a-GCST005581'))
# 2. Extract outcome data (GWAS ID example: ebi-a-GCST90025981)
outcome_data <- extract_outcome_data(
   snps = exposure_data$SNP,
   outcomes = c('ebi-a-GCST90014122'),
   proxies = 1,
   rsq = 0.8,
   align_alleles = 1,
   palindromes = 1,
   maf_threshold = 0.3
)

# 3. Data harmonization
harmonized_data <- harmonise_data(
   exposure_dat = exposure_data,
   outcome_dat = outcome_data
)


# 4. Save harmonized data
write.csv(harmonized_data, file = "harmonise", row.names = FALSE)

# 5. Run main MR analysis (default methods include IVW, Egger, Weighted Median, etc.)
mr_results <- mr(harmonized_data)
mr_results
# 6. Calculate OR values and save
or_results <- generate_odds_ratios(mr_results)
or_results
write.csv(or_results, file = "odds_ratios.csv", row.names = TRUE)
```

```
# 7. View available MR methods
mr_method_list()

# 8. Optional: Run with custom MR methods
# mr_results_custom <- mr(harmonized_data,
#                         method_list = c("mr_ivw",
#                                         "mr_egger_regression",
#                                         "mr_weighted_median",
#                                         "mr_simple_mode",
#                                         "mr_weighted_mode"))

# 9. Heterogeneity test
heterogeneity_results <- mr_heterogeneity(harmonized_data)
heterogeneity_results
write.csv(heterogeneity_results, file = "mr_heterogeneity.csv", row.names = TRUE)

# 10. Outlier detection (MR-PRESSO)
presso_results <- run_mr_presso(harmonized_data, NbDistribution = 1000)
presso_results
write.csv(presso_results, file = "run_mr_presso.csv", row.names = TRUE)

# 11. Horizontal pleiotropy test (e.g., MR-Egger intercept)
pleiotropy_results <- mr_pleiotropy_test(harmonized_data)
pleiotropy_results
write.csv(pleiotropy_results, file = "mr_pleiotropy_test.csv", row.names = TRUE)

# 12. Single SNP analysis
singlesnp_results <- mr_singlesnp(harmonized_data)
write.csv(singlesnp_results, file = "res_single.csv", row.names = TRUE)

# 13. Leave-one-out analysis (LOO)
loo_results <- mr_leaveoneout(harmonized_data)

# 14. Plotting

## Scatter plot
res_scatter <- mr_scatter_plot(mr_results, harmonized_data)
res_scatter[[1]]
library(ggplot2)
ggsave(res_scatter[[1]], file="res_scatter.pdf", width=7, height=7)


## Forest plot
res_forest <- mr_forest_plot(singlesnp_results)
```

```
res_forest[[1]]
ggsave(res_forest[[1]], file="res_forest.pdf", width=7, height=7)

## Leave-one-out plot
res_loo <- mr_leaveoneout_plot(loo_results)
res_loo[[1]]
ggsave(res_loo[[1]], file="res_loo.pdf", width=7, height=7)


## Funnel plot
res_funnel <- mr_funnel_plot(singlesnp_results)
res_funnel[[1]]
ggsave(res_funnel[[1]], file="res_funnel.pdf", width=7, height=7)


# 15. Perform Steiger filtering to test directionality

# 1. Extract exposure data (PBC)
pbc_exp_dat <- extract_instruments('ebi-a-GCST005581')

# 2. Extract outcome data (lacunar stroke)
lac_out_dat <- extract_outcome_data(
  snps = pbc_exp_dat$SNP,
  outcomes = 'ebi-a-GCST90014122'
)

# 3. Harmonise data
dat <- harmonise_data(pbc_exp_dat, lac_out_dat)

out <- directionality_test(dat)
# 5. View and save results
print(out)
write.csv(out, file = "mr_steiger_test.ebi-a-GCST005581.ebi-a-GCST90014122.csv", row.names = FALSE)
```
